# Crystal Structure of the Human Astrovirus Capsid Protein

**DOI:** 10.1128/JVI.00694-16

**Published:** 2016-09-29

**Authors:** Yukimatsu Toh, Justin Harper, Kelly A. Dryden, Mark Yeager, Carlos F. Arias, Ernesto Méndez, Yizhi J. Tao

**Affiliations:** a Department of BioSciences, Rice University, Houston, Texas, USA; b Department of Molecular Physiology and Biological Physics, University of Virginia School of Medicine, Charlottesville, Virginia, USA; c Departamento de Genética del Desarrollo y Fisiología Molecular, Universidad Nacional Autonoma de México, Cuernavaca, Morelos, Mexico; University of Pittsburgh School of Medicine

## Abstract

Human astrovirus (HAstV) is a leading cause of viral diarrhea in infants and young children worldwide. HAstV is a nonenveloped virus with a T=3 capsid and a positive-sense RNA genome. The capsid protein (CP) of HAstV is synthesized as a 90-kDa precursor (VP90) that can be divided into three linear domains: a conserved N-terminal domain, a hypervariable domain, and an acidic C-terminal domain. Maturation of HAstV requires proteolytic processing of the astrovirus CP both inside and outside the host cell, resulting in the removal of the C-terminal domain and the breakdown of the rest of the CP into three predominant protein species with molecular masses of ∼34, 27/29, and 25/26 kDa, respectively. We have now solved the crystal structure of VP90^71–415^ (amino acids [aa] 71 to 415 of VP90) of human astrovirus serotype 8 at a 2.15-Å resolution. VP90^71–415^ encompasses the conserved N-terminal domain of VP90 but lacks the hypervariable domain, which forms the capsid surface spikes. The structure of VP90^71–415^ is comprised of two domains: an S domain, which adopts the typical jelly-roll β-barrel fold, and a P1 domain, which forms a squashed β-barrel consisting of six antiparallel β-strands similar to what was observed in the hepatitis E virus (HEV) capsid structure. Fitting of the VP90^71–415^ structure into the cryo-electron microscopy (EM) maps of HAstV produced an atomic model for a continuous, T=3 icosahedral capsid shell. Our pseudoatomic model of the human HAstV capsid shell provides valuable insights into intermolecular interactions required for capsid assembly and trypsin-mediated proteolytic maturation needed for virus infectivity. Such information has potential applications in the development of a virus-like particle (VLP) vaccine as well as small-molecule drugs targeting astrovirus assembly/maturation.

**IMPORTANCE** Human astrovirus (HAstV) is a leading cause of viral diarrhea in infants and young children worldwide. As a nonenveloped virus, HAstV exhibits an intriguing feature in that its maturation requires extensive proteolytic processing of the astrovirus capsid protein (CP) both inside and outside the host cell. Mature HAstV contains three predominant protein species, but the mechanism for acquired infectivity upon maturation is unclear. We have solved the crystal structure of VP90^71–415^ of human astrovirus serotype 8. VP90^71–415^ encompasses the conserved N-terminal domain of the viral CP. Fitting of the VP90^71–415^ structure into the cryo-EM maps of HAstV produced an atomic model for the T=3 icosahedral capsid. Our model of the HAstV capsid provides valuable insights into intermolecular interactions required for capsid assembly and trypsin-mediated proteolytic maturation. Such information has potential applications in the development of a VLP vaccine as well as small-molecule drugs targeting astrovirus assembly/maturation.

## INTRODUCTION

Members of the Astroviridae family possess a nonsegmented, positive-sense, single-stranded RNA (ssRNA) genome with a nonenveloped icosahedral capsid (1). Astroviruses are organized into two genera, Mamastrovirus and Avastrovirus, that infect mammals and avian species, respectively. Astrovirus was first detected in 1975 in fecal samples collected from infants, wherein viral particles were found to display a star-like morphology by negative-staining transmission electron microscopy (EM) (2, 3). Human astrovirus is considered one of the major causes of childhood viral gastroenteritis worldwide (4). Human astrovirus (HAstV) can be further divided into eight major serotypes, with HAstV-1 (human astrovirus serotype 1) being the predominant clinical isolate (5). Transmission of human astrovirus occurs through the ingestion of contaminated food or water, leading to infection of gut epithelial cells via receptor-mediated endocytosis, which ultimately culminates in the lytic release of viral progenies (6, 7).

**FIG 1:**
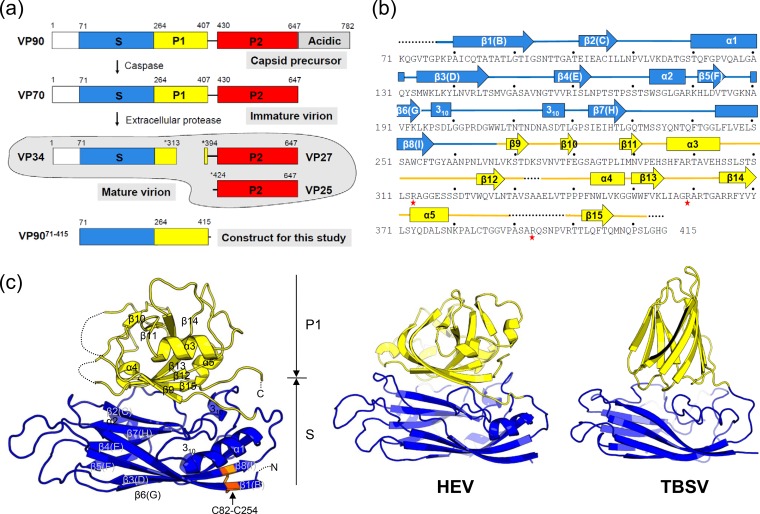
Astrovirus CP. (a) Proteolytic processing of astrovirus VP90. Astrovirus VP90 consists of a conserved region (white, blue, and yellow), a variable region (red), and an acidic C-terminal region (gray). Stars represent trypsin digestion sites. (b) Secondary-structure assignment of VP90^71–415^. α-Helices are indicated by tubes, β-strands are indicated by arrows, loops are indicated by thick lines, and disordered regions are indicated by dotted lines. Stars highlight trypsin digestion sites confirmed previously and in this study. (c) Crystal structure of VP90^71–415^. The molecule is colored with the S domain in blue and the P1 domain in yellow. The disulfide bond C82-C254 is highlighted in orange. The equivalent parts of the HEV CP and the TBSV CP are shown on the right.

The ∼7-kb genomic RNA of human astrovirus is polyadenylated and contains three open reading frames (ORFs) (8, 9). ORF1a and the downstream overlapping ORF1b encode two nonstructural polyproteins, nsp1a and nsp1ab, that are proteolytically processed into smaller proteins implicated in viral genome replication (8–13). At the 3′ end of the genome, ORF2 encodes the capsid protein (CP), which is translated from a subgenomic RNA, thus allowing for the differential regulation of structural and nonstructural protein synthesis (14). The astrovirus CP, which is initially synthesized as VP90, can be divided into three distinct domains: a conserved amino terminus (amino acids [aa] 1 to 415 of VP90 [VP90^1–415^]), a hypervariable central region (VP90^416–646^), and an acidic C-terminal domain (VP90^647–782^) (15, 16) (Fig. 1a). The acidic C-terminal domain mediates a transient association between full-length VP90 and host membranous structures via an endoplasmic reticulum (ER)-targeting motif, allowing for the colocalization of capsid assembly and viral genome replication (17, 18). Upon assembly of the VP90 capsid lattice, the acidic C-terminal domain is cleaved intracellularly by host caspases, leading to its exclusion from the virion and the conversion of VP90 to VP70 (19, 20) (Fig. 1a). When overexpressed in eukaryotic hosts, the astrovirus CP, in the form of either VP90 or VP70, was able to self-assemble into virus-like particles (VLPs) (21–23).

After astrovirus particles are released from infected cells, further proteolytic processing of the viral capsid by host extracellular proteases is required for virus infectivity. In cell culture, the inclusion of trypsin is essential for the successful propagation of human astrovirus (24). Trypsin treatment produces a capsid composed of three predominant protein species with molecular masses of ∼34 kDa (VP34), 27/29 kDa (VP27/29), and 25/26 kDa (VP25/26) (14, 19, 23, 25, 26). VP34 is derived from the conserved N-terminal domain (VP90^1–415^), which comprises the capsid shell (i.e., the continuous, spherical capsid), whereas both VP27/29 and VP25/26 are from the hypervariable region with a different N terminus, which forms the capsid surface spike (15, 27) (Fig. 1a). Within the conserved N-terminal domain, the polypeptide spanning residues 1 to 70 is an RNA coordination motif that is enriched in basic amino acids, likely structurally disordered, and dispensable for particle assembly (21), a situation similar to that of many small RNA plant viruses such as tomato bushy stunt virus (TBSV) and turnip crinkle virus (28, 29).

The astrovirus capsid is an external structural barrier that not only encapsidates nucleic acids but also interacts with the host to define cell tropism, mediate cell entry, and trigger the host immune response (1). A 25-Å-resolution cryo-EM reconstruction of an immature astrovirus capsid shows T=3 icosahedral symmetry with a total of 90 spikes distributed along the 2-fold as well as the pseudo-2-fold symmetry axes (30). In comparison, the reconstruction of a mature virion shows an overall similar capsid topology, but only 30 spikes are observed along icosahedral 2-fold symmetry axes (30). The crystal structure of the human astrovirus capsid spike has also been determined as dimers (15). The overall structure of each spike/projection domain has a unique three-layered β-sandwich fold, with a core, six-stranded β-barrel structure that is also found in hepatitis E virus (HEV) capsid protrusions (31, 32).

To further enhance our understanding of astrovirus assembly and maturation, here we report the crystal structure of VP90^71–415^ from HAstV-8 at a 2.15-Å resolution. VP90^71–415^, which covers the conserved N-terminal region of VP70 except for the putative RNA coordination motif (i.e., aa 1 to 70), was crystallized as monomers with 1 molecule per crystallographic asymmetric unit (CAU). The structure of VP90^71–415^ shows two domains: an S domain that adopts the typical jelly-roll β-barrel fold and a P1 domain that has the appearance of a squashed β-barrel consisting of six antiparallel β-strands. A Dali search indicated that VP90^71–415^ is a close structural homolog of the HEV CP in spite of the lack of any detectable sequence similarity (31, 32). By fitting the VP90^71–415^ crystal structure into the available cryo-EM maps (30), an atomic model of the astrovirus capsid is derived, which highlights important molecular interactions involved in the formation of various types of capsomeres found in a T=3 icosahedral capsid. The VP90^71–415^ structure also provides insights into the trypsin-mediated capsid maturation process and the accompanying structural changes that lead to enhanced viral infectivity.

## MATERIALS AND METHODS

### Subcloning, protein expression, and purification.

The coding sequence for HAstV-8 CP^71–415^ (strain Yuc8) (GenBank accession number AF260508) was cloned between the NcoI and HindIII sites of the vector pET28a (Novagen) by using forward primer 5′-GGCGCCCATGGAAAAACAAGGTGTCACAGGACCAAAACCT-3′ and backward primer 5′-GCGCCAAGCTTTTAGTGATGATGATGATGATGACCACCACCATGACCTAAACTAGGCTGATTCATC-3′.

A 6×His tag and a GGG linker sequence were engineered at the C terminus of the construct to facilitate protein purification. Recombinant protein was expressed in Escherichia coli by using the Rosetta 2(DE3) strain. Cells were first grown to an optical density at 600 nm (OD_600_) of 0.8 to 1.0 at 37°C and then induced with 1 mM IPTG (isopropyl-β-d-1-thiogalactopyranoside) for 19 h at 30°C. Cells were harvested and lysed by using a sonicator (Branson 250 Sonifier) for 15 min at 4°C. The lysis buffer consisted of 300 mM NaCl, 50 mM Tris (pH 7.5), 5 mM 2-mercaptoethanol, 5 mM imidazole, 1 mM PMSF (phenylmethylsulfonyl fluoride), 0.5 μg/ml pepstatin, and 0.5 μg/ml leupeptin. The lysate was clarified by centrifugation at 12,000 × *g* at 4°C for 45 min. The supernatant was subjected to affinity purification using Ni-nitrilotriacetic acid (NTA) resin (Thermo Scientific). Bound protein was eluted by using an elution buffer containing 500 mM NaCl, 50 mM Tris (pH 7.5), 5 mM 2-mercaptoethanol, and 300 mM imidazole. Recombinant protein was further purified by ion exchange chromatography using a HiTrap Q HP column (GE Healthcare) and by size exclusion chromatography using a HiLoad Superdex 200 16/60 gel filtration column (GE Healthcare). The final protein was at least 95% pure as judged by SDS-PAGE. The protein concentration was determined by the *A*_280_ reading from a NanoDrop 2000/2000c spectrophotometer (Thermo Scientific). The molar extinction coefficient (ε) of CP^71–415^ was calculated to be 1.58 by the ExPASy ProtParam tool. Approximately 5 mg of purified protein could be obtained from 6 liters of cell culture.

To obtain selenomethionine (SeMet)-labeled proteins, VP90^71–415^ was overexpressed by using M9 minimal medium containing SeMet and a mixture of six other amino acids to prevent methionine synthesis (33). SeMet-labeled proteins were purified by using the same protocol as the one described above.

### Crystallization and structure determination.

Purified HAstV-8 VP90^71–415^ proteins, both native and SeMet-labeled forms, were concentrated to 10 mg/ml and subjected to crystallization screening. The best crystals were obtained by the hanging-drop vapor diffusion method at 25°C by mixing 2 μl of the protein solution with an equal volume of reservoir solution containing 0.2 M ammonium phosphate and 22% polyethylene glycol 4000 (PEG 4000). For data collection, crystals were briefly soaked in a cryoprotectant made from mother liquor supplemented with 23% (vol/vol) glycerol and flash-cooled in a nitrogen cryostream. Diffraction data were collected at the Advanced Photon Source (APS) (Argonne National Laboratory, Chicago, IL, USA) Life Sciences Collaborative Access Team (LS-CAT) F line. For SeMet-labeled crystals, a single-wavelength anomalous dispersion (SAD) data set of 720 frames was collected at the peak wavelength for Se (0.97872 Å) by using a detector-to-crystal distance of 200 mm, an exposure time of 2 s, and an oscillation angle of 1°. All of the data were processed by using the HKL2000 program (34).

The structure of VP90^71–415^ was determined to a 2.15-Å resolution by Se-SAD. The AutoSol program in PHENIX (35) located four out of the six Se atoms in the asymmetric unit. Model building was carried out by using the program AutoBuild in PHENIX and Coot (36). Structure refinement was performed by using the maximum likelihood method with the phenix.refine program from the PHENIX suite (35). The data statistics are summarized in Table 1.

**TABLE 1:**
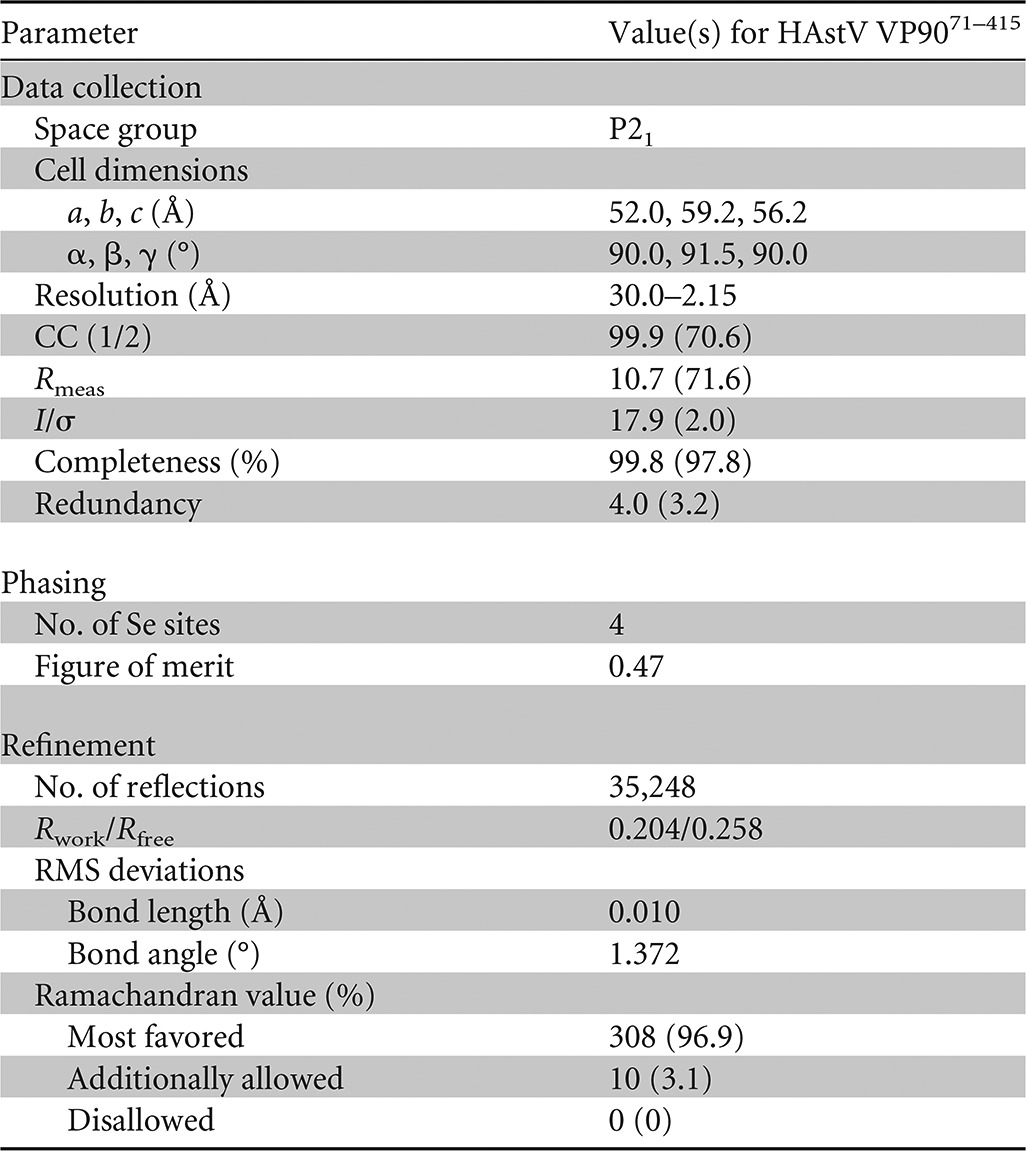
X-ray data statistics^*a*^

a Statistics in parentheses refer to the outer-resolution shell. CC (1/2), percentage of correlation between intensities of random half-data sets; RMS, root mean square.

### HAstV-8 VP90^71–415^ structure fitted into cryo-EM structures.

Pseudoatomic models for the astrovirus capsid were generated by fitting the crystal structure of VP90^71–415^ into the HAstV cryo-EM reconstruction maps calculated to a 25-Å resolution (30). Three copies of VP90^71–41^ were manually fitted into a region of the cryo-EM maps corresponding to an icosahedral asymmetric unit using the UCSF Chimera program (37). Fitting was further improved by using the “Fit in Map” option in Chimera. A correlation coefficient of 0.90 was given for the combined S, P1, and P2 domains. The entire T=3 capsid model was generated by applying icosahedral symmetry. This model was used for studying CP capsomere interactions.

Cartoon and surface representations were generated with the PyMOL (http://www.pymol.org/) and UCSF Chimera programs, respectively.

### Accession number(s).

Atomic coordinates and structure factors have been deposited in the RCSB Protein Data Bank (PDB) under accession number 5IBV.

## RESULTS AND DISCUSSION

### Structure of astrovirus VP90^71–415^.

Three truncation mutants of HAstV-8 VP90, including VP90^71–415^ (38.1 kDa; pI 9.49), VP90^71–313^ (26.8 kDa; pI 8.45), and VP90^71–283^ (23.51 kDa; pI 8.45), were cloned and expressed in E. coli (Fig. 1a). Residues 71 and 415 have been mapped to roughly the beginning of the S domain and the end of the P1 domain, respectively (15, 22, 23, 27). Therefore, VP90^71–415^ was expected to contain both the S and P1 domains of the astrovirus CP, but VP90^71–283^ should contain only the S domain. VP90^71–313^ was designed to mimic VP34, the longest peptide fragment observed in mature astrovirus after trypsin activation (23). All three constructs were expressed as soluble proteins. Furthermore, gel filtration chromatography using a HiLoad Superdex 200 16/60 gel filtration column (S200) showed that the three proteins were all eluted as a single peak with an apparent molecular mass of ∼30 kDa, indicating the formation of monomers in solution. Therefore, the S or S-P1 domain alone appeared to be insufficient to mediate the assembly of high-order oligomers/capsomeres.

Among the three constructs, only VP90^71–415^ produced single crystals. The space group was determined to be P2_1_ with *a* equal to 52.0 Å, *b* equal to 59.2 Å, *c* equal to 56.2 Å, and β equal to 91.5°. The structure was determined to a 2.15-Å resolution by Se-SAD (Fig. 1 and Table 1). There is one VP90^71–415^ molecule in each crystallographic asymmetric unit cell, consistent with VP90^71–415^ being a monomer in solution. The final model, which was refined to an *R*_work_ value of 0.204 and an *R*_free_ value of 0.258, contains 321 out of 345 residues in total. No density was observed for the initial Met residue and the 6×His tag. Additional disordered regions include residues 71 to 76, 332 to 334, 390 to 398, and 413 to 415 (Fig. 1b and c). An intramolecular disulfide bond is formed between C82 and C254 (Fig. 1c).

The structure of VP90^71–415^ is organized into two domains called the S domain (residues 71 to 256) and the P1 domain (residues 257 to 415) (Fig. 1b and c). The S domain has the typical jelly-roll β-barrel fold with eight antiparallel β-strands that is broadly conserved among many viral capsid proteins. These eight β-strands, often designated by the letters B to I, form two twisted β-sheets called BIDG and CHEF (Fig. 1b). The surfaces of the two β-sheets are decorated by a number of loops and also four helices (i.e., α1 from the CD loop, α2 from the EF loop, and two 3_10_ helices from the GH loop). The P1 domain has an antiparallel β-barrel structure composed of seven β-strands (i.e., β9 to β15) and three α-helices (i.e., α3 to α5). The P1 domain is connected to the S domain through an asparagine-rich linker loop (i.e., residues 257 to 267). A substantial amount of surface area (∼2,400 Å^2^) is buried between the S and P1 domains. This interaction, which is mostly hydrophobic in nature, is mediated by β(C)2(C) and the CD loop, EF loop, and GH loop from the S domain and the domain linker loop, β9, β12, β13, β15, α4, and α5 from the P1 domain. The C terminus of the P1 domain is located externally near the S-P1 domain interface. In the astrovirus capsid, the C terminus of P1 is expected to connect to the P2 domain, which forms the dimeric spikes on the outer surface of the viral capsid (15).

### Structural comparison with other viral CPs.

A structural homolog search using the Dali server (38) showed that the S domain of VP90^71–415^ was best aligned to the jelly-roll domain of carnation mottle virus (CMV) (*Z* score of 17.8, with a value of >2.0 being significant) (39), TBSV (*Z* score = 16.0) (40), ryegrass mottle virus (RMV) (*Z* score = 15.8) (41), Orsay virus (OV) (*Z* score = 15.7) (42), and HEV (*Z* score = 15.6) (31, 32). When the P1 domain was used as the reference for the Dali search, the hit with the highest *Z* score was HEV (*Z* score = 7.0). When both the S and P1 domains of VP90^71–415^ were used as the search model, HEV came up with the highest *Z* score, 27.0, which was followed by CMV (*Z* score = 16.0), TBSV (*Z* score = 14.4), RMV (*Z* score = 14.0), and OV (*Z* score = 13.8) (Fig. 1c). The finding that HEV persistently showed up as a top structural homolog based on either individual domains or the entire structure indicates that human astrovirus and HEV are evolutionarily related, consistent with previous conclusions based on structural comparison using the P2 domain alone (15, 30).

### T=3 HAstV-8 capsid models.

Structures of both immature human astrovirus (HAstV-8) (EMD-5414) and mature human astrovirus (HAstV-1) (EMD-5413) have been established by cryo-EM reconstruction to a 25-Å resolution (30). Therefore, the structure of VP90^71–415^ allowed us to build an atomic model of the astrovirus capsid. Together with the crystal structure of the capsid spike (15), we now have atomic coordinates for the entire astrovirus VP70 protein except for the RNA coordination motif, which is expected to be mostly structurally disordered.

**FIG 2:**
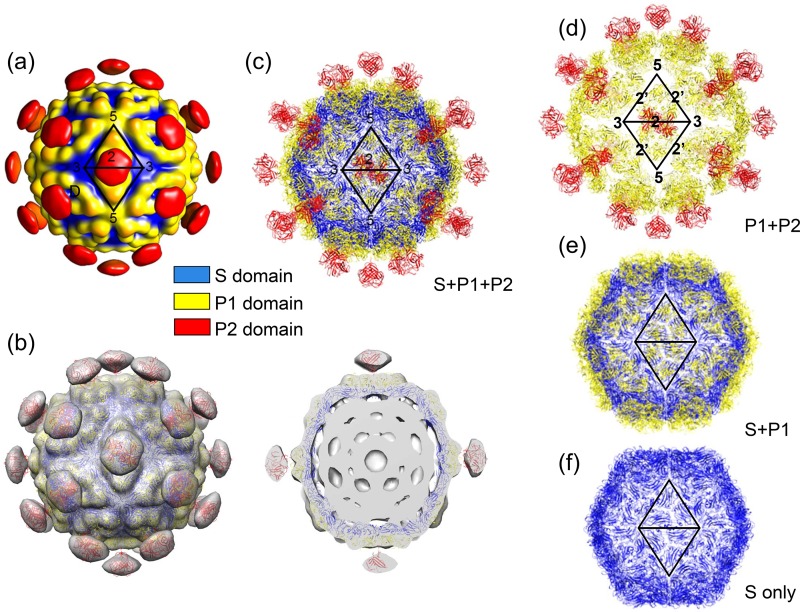
Human astrovirus capsid. (a) Cryo-EM structure of mature HAstV-1. The surface is colored by radial depth cue from blue to red. The colors blue, yellow, and red roughly match the S, P1, and P2 domains of the astrovirus CP, respectively. (b) Astrovirus capsid model docked into the three-dimensional cryo-EM density map of mature HAstV-1. (Left) Outside view; (right) central slab. For the capsid model, the S, P1, and P2 domains are colored according to the color key. (c) Atomic model of the astrovirus capsid by EM docking. The S, P1, and P2 domains are colored according to the color key. (d) Astrovirus capsid with only the P1 and P2 domains. (e) Astrovirus capsid with the S and P1 domains. (f) Mature astrovirus capsid with only the S domain. Two asymmetric units are highlighted by two triangles panels a and c to f. Icosahedral symmetry axes (2 for 2-fold, 3 for 3-fold, 5 for 5-fold, and 2′ for quasi-2-fold) are also highlighted where space is available.

Comparison of the structures of the immature and mature astrovirus capsids shows a major difference in the stoichiometry of the surface spike: while the immature astrovirus capsid shows 90 dimeric spikes along the icosahedral and quasi-2-fold symmetry axes, the mature astrovirus capsid shows only 30 spikes along 2-fold symmetry axes (Fig. 2a) (30). The crystal structure of VP90^71–415^ fits well into the EM maps of the immature and mature astrovirus capsids, producing nearly identical models. Due to the lack of a meaningful difference, only the mature capsid model is presented (Fig. 2a and b). Although the available EM map for the mature astrovirus capsid is for HAstV-1, and our crystal structure is for HAstV-8, the structural difference should not be a concern at this resolution, as the sequences of HAstV-1 and HAstV-8 are 83% identical in the conserved N-terminal region of the CP (i.e., aa 1 to 415). The outer diameter of our mature astrovirus model without the surface spikes is ∼350 Å. The S domain assembles into a continuous capsid shell, while the P1 domain forms trimeric clusters on the capsid surface (Fig. 2c, e, and f). These P1 trimeric clusters are in close contact across the icosahedral 2-fold symmetry axes, but the P1 trimeric clusters related by pseudo-2-fold symmetry axes do not interact, resulting in the breakdown of pseudo-6-fold symmetry on 3-fold icosahedral symmetry axes (Fig. 2d and e). Small depressions are observed at both 5-fold and 3-fold symmetry axes.

**FIG 3:**
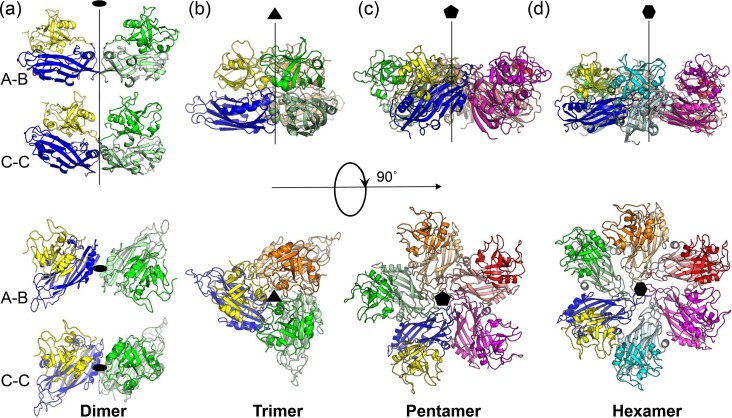
Capsomeres from the T=3 mature astrovirus capsid model. (a) Dimers. C-C and A-B dimers are related by icosahedral and quasi-2-fold symmetry axes, respectively. (b) Trimer. (c) Pentamer. (d) Hexamer. Two-, 3-, 5-, and 6-fold symmetry axes are represented by black lines and highlighted by an oval, triangle, pentagon, and hexagon, respectively. The molecules in the top row are viewed from the side, while the molecules in the bottom row are viewed along the symmetry axes. A reference molecule is colored with the S domain in blue and the P1 domain in yellow. Other symmetry-related molecules are each shown in a different color, with the S domain and P1 domain from the same molecule shown in lighter and darker shades of the same colors, respectively.

**FIG 4:**
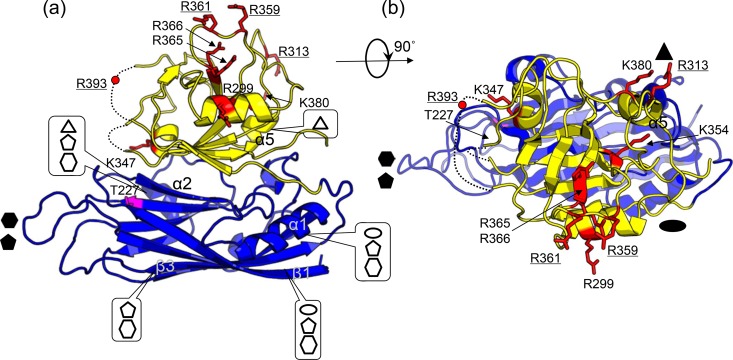
VP90^71–415^ molecule highlighting capsomere interactions and trypsin cleavage sites. (a) Side view. The viewing orientation is the same as that described in the legend of Fig. 1c. (b) Top view at a 90° rotation from the view in panel a. The S domain is in blue, and the P1 domain is in yellow. Residues implicated in trypsin cleavage are highlighted in red, with side chains shown in a stick-and-ball representation. Likely cleavage sites as discussed in the text are underlined. Secondary-structure elements involved in capsomere interactions are pinpointed by ovals (2-fold), triangles (3-fold), pentagons (5-fold), and hexagons (6-fold).

**FIG 5:**
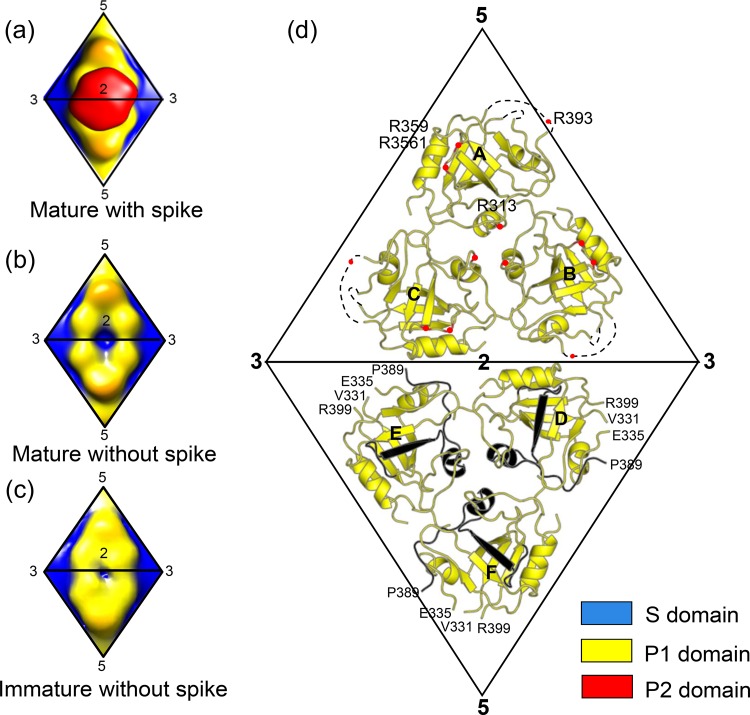
Trypsin-mediated human astrovirus capsid maturation. (a) EM reconstruction of the mature capsid with a surface spike. (b) EM reconstruction of the mature capsid without a spike. (c) EM reconstruction of the immature capsid without a spike. Molecules A-C and D-F form two separate trimers across the 2-fold symmetry axis. In panels a to c, only two asymmetry units are shown. (d) Fitted capsid structure model. The P1 domain is in yellow. Trypsin cleavage sites are highlighted in the top asymmetric unit. In the asymmetric unit at the bottom, residues 360 to 398 are highlighted in dark gray.

Without the surface spike, the VP90^71–415^ dimer buries the smallest amount of surface areas among all types of capsomeres found in the T=3 capsid. There are two types of dimers: one that is relatively flat, sitting on 2-fold axes (i.e., C-C dimer), and another that has an inwardly bent conformation, located on quasi-2-fold axes (i.e., A-B dimer). Due to the different bending angles, we observed dramatic differences in the gap distances between the two P1 domains in the two types of dimers, with ∼5 Å for the C-C dimer and ∼25 Å for the A-B dimer (Fig. 3a). Consequently, the two P1 domains from the A-B dimer are completely segregated. The A-B dimer interface buried a total surface area of only ∼300 Å^2^, but nearly ∼1,200 Å^2^ of surface area is buried in the C-C dimer. Previous studies of other T=3 viral capsids indicate that the different bending angles observed in A-B and C-C dimers could be maintained by different viral nucleic acid binding modes and/or differentially ordered structural elements from the CP N-terminal sequence at the icosahedral versus quasi-2-fold symmetry axes (28). The secondary structural elements α1 and β1 from the S domain are found at the interface of both dimers (Fig. 3a to 5). The C-C dimer interface has additional contacts made by the loop connecting the S and P1 domains.

Our fitted model of the mature astrovirus capsid shows that the VP90^71–415^ trimer buries a substantial amount of surface (i.e., ∼2,000 Å^2^) between adjacent subunits (Fig. 3b and 4). The interaction is mediated largely by the following structural elements: (i) helix α2 and the GH loop from the S domain, (ii) helix α5 and an extended loop (residues 305 to 324) connecting α3 and β12 from the P1 domain, and (iii) the loop connecting the S and P domains (Fig. 4). In particular, the 3-fold symmetry axis is surrounded by the GH loop from the S domain, helix α5, and the loop (residues 305 to 324) connecting α3 and β12 from the P1 domain (Fig. 4).

The astrovirus VP90^71–415^ pentamers are maintained exclusively by interactions mediated by the S domain (Fig. 3c and 4). The 5-fold symmetry axis is surrounded by the ED, FG, and HI loops. Additionally, helix α1 and the BIDG β-sheet from one subunit make contact with helix α2 and the CHEF β-sheet from the adjacent subunit in the same pentamer. The total buried surface area between adjacent subunits is ∼2,000 Å^2^ in pentamers. Matsui et al. reported previously that mutations at T227 resulted in the disruption of capsid assembly in HAstV-1 (43). Our structural model shows that T227 is from β7(H) located at the pentamer interface (Fig. 4).

Close inspection of the VP90^71–415^ hexamer shows that the S domain makes interactions similar to those in the pentamer (Fig. 3d and 4). The major difference is observed in the P1 domain. While the five P1 domains from a pentamer are completely isolated from each other, the six P1 domains in a hexamer interact with each other in pairs (e.g., molecule 1 interacts with molecule 2, molecule 3 interacts with molecule 4, and molecule 5 interacts with molecule 6). The interface between two interacting P1 domains is mediated by helices α3 and α4. The total buried surface areas are ∼2,000 Å^2^ for adjacent subunits with noninteracting P1 domains and ∼2,100 Å^2^ for adjacent subunits with interacting P1 domains. It is worthwhile to note that some structural clashes are observed in both the pentamer and hexamer near the 5- and 6-fold symmetry axes, suggesting that the three structured loops ED, FG, and HI from the S domain may adopt a somewhat different conformation upon the assembly of a capsid.

### Mapping of trypsin cleavage sites required for astrovirus maturation.

Proteolytic cleavage is a common activation mechanism for both enveloped and nonenveloped viruses. A recurring theme is that proteolytic cleavage either releases cell penetration factors or triggers conformational changes in the capsid or cell attachment proteins (44–47). Astrovirus infectivity is also dependent on proteolysis-mediated maturation (24). Upon activation by trypsin treatment, VP70 from the immature particle is converted to several smaller polypeptide species, including VP34, VP27, and VP25 (14, 19, 23, 25, 26).

**FIG 6:**
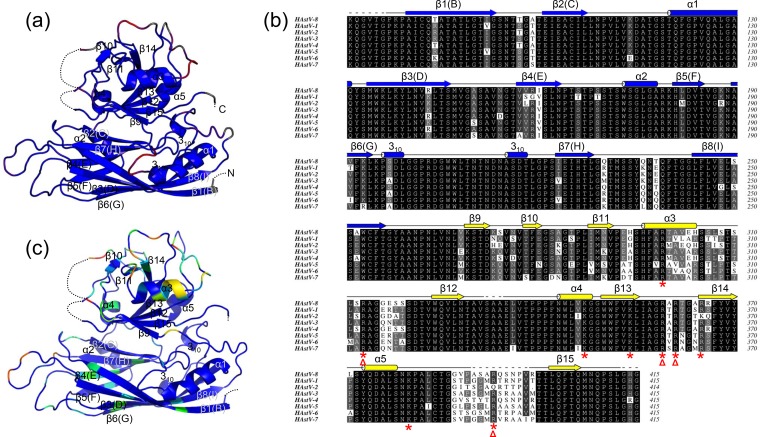
Structural conservation among the eight HAstV serotypes. (a) Structural comparison between HAstV-1 and HAstV-8 VP90^71–415^. Variable regions are shown in red, while regions superimposed well are shown in blue. (b) Multiple-sequence alignment for CPs from the eight astrovirus serotypes HAstV-1 (UniProtKB accession number O12792), HAstV-2 (UniProtKB accession number J7LPD5), HAstV-3 (UniProtKB accession number Q9WFZ0), HAstV-4 (UniProtKB accession number Q3ZN05), HAstV-5 (UniProtKB accession number Q4TWH7), HAstV-6 (UniProtKB accession number Q67815), HAstV-7 (UniProtKB accession number Q96818), and HAstV-8 (GenBank accession number AAF85964.1). Only residues 71 to 415 of the CP are included in the alignment. Superimposed secondary structural elements are taken from the crystal structure of HAstV-8 VP90^71–415^. Conserved arginines and lysines from the P1 domain are highlighted by red stars, with likely cleavage sites further underlined by red triangles. (c) Sequence variations mapped to the HAstV-8 VP90^71–415^ structure. The structure is colored in rainbow coloring, with conserved regions in blue and poorly conserved regions in red.

With the crystal structure of VP90^71–415^ solved, we are able to map the terminal ends of VP34 and VP27, two of the three major peptide fragments from mature astrovirus, in the context of the capsid model. The N-terminal residue of HAstV-8 VP27 was previously determined to be Q394 by N-terminal sequencing (23), and the structure of VP90^71–415^ shows that R393, which is strictly conserved in all human astrovirus serotypes (Fig. 6b), is located at a structurally disordered surface loop (i.e., ^390^ASARQSNPV^398^) facing the surface depression on 5-fold and quasi-6-fold symmetry axes (Fig. 4 and 5) and is completely solvent exposed. In HAstV-2, the 26-kDa protein was presumably generated by a similar cleavage event at Arg394 at an equivalent position (26). The ^390^ASARQSNPV^398^ loop is connected at its C terminus to β15, which is part of a four-stranded β-sheet (i.e., made of β13, β12, β15, and β9) that closely interacts with the S domain from the opposing side. Therefore, in principle, VP27 could remain tethered to the capsid through the β-sheet interaction mediated by β15. In comparison, VP25 starts its N terminus at residue 424, which is beyond the P1 domain, and therefore would lose its grip on the capsid after its cleavage (15, 30).

VP34 has an intact N terminus, but its C terminus has not yet been experimentally defined. The apparent molecular mass of 34 kDa suggests that its C terminus is likely to end at around residue 310. Examination of the protein sequence shows three trypsin-susceptible sites around this area, including R299, R313, and K347. Because R299 and K347 are located in the α3 and α4 helices, respectively, the most likely cleavage site is R313, which is situated in a flexible loop located on the surface of the P1 domain facing the quasi-3-fold symmetry axis (Fig. 4 and 5). Multiple-sequence alignment shows that R313 is strictly conserved in all astrovirus serotypes (Fig. 6b). The calculated molecular mass of the polypeptide containing residues 1 to 313 is 33.7 kDa, which closely matches that of VP34 (19, 21).

### Implications for astrovirus maturation.

Previous work by Méndez et al. indicated that VP34 is generated by the progressive shortening of VP41 (containing residues 1 to 393, with a theoretical molecular mass 42.9 kDa) through a number of intermediates, including VP38.5 (23). Why is a cascade of trypsin cleavage events required to activate virus infectivity? The sequential order of the observed trypsin cleavages may imply that the final cleavage site needed to activate virus infectivity is not accessible at the beginning, but the initial cleavages could induce structural changes to expose the R313 site. Indeed, our capsid model shows that R313 is partially buried near the quasi-3-fold symmetry axes (Fig. 5d). While we cannot rule out the possibility that the α3-β12 loop hosting R313 could undergo some local structural rearrangements upon capsid formation, it is highly likely that R313 is not fully revealed in the context of the capsid without the previous cuts by trypsin.

Another interesting question is what happens to the polypeptide region from residue 314 to 393 after trypsin treatment. This peptide, which covers the region from after the C terminus of VP34 to the N terminus of VP27, has a calculated molecular mass of 8.5 kDa. The VP90 polypeptide region from residues 314 to 393 contains a total of six arginines/lysines (i.e., K347, R359, R361, R365, R366, and K380) that are potentially susceptible to trypsin. These six residues are all surface exposed in the crystal structure of VP90^71–415^. In the context of the capsid, the most susceptible sites are R359 and R361, which are located in a structurally flexible loop hovering over the P1 domain (Fig. 4 and 5). The accessibility of the other four residues can be ranked in descending order as R365/R366 > K347 > K380 due to the following considerations: R365 and R366 are in a β-strand underneath the loop containing R359 and R361, K347 is in helix α4 adjacent to the continuous capsid shell, and K380 lies at the quasi-3-fold trimer interface. Indeed, the theoretical molecular mass of the polypeptide containing residues 1 to 359 is 38.5 kDa, exactly matching that of the cleavage intermediate VP38.5 (23), suggesting a cleavage event at either R359 or R361 during astrovirus maturation. In our capsid structure model, the polypeptide spanning residues 359 to 393, which is comprised of β14 and α5, is located near the center of the quasi-3-fold trimers (Fig. 5d).

The proteolytically induced maturation of the capsid results in a >100-fold increase in infectivity (23, 24). The underlying mechanism of acquired infectivity has not yet been determined but could be related to an altered structural stability of the capsid that is essential for uncoating, exposure of cell receptor binding sites, and/or liberation of viral penetration factors residing within the cleaved C terminus of the VP41 intermediate that would allow astrovirus internalization (23). The capsid model of VP90^71–415^ will provide a valuable structural framework for further biochemical and genetic analyses to pinpoint cleavage sites essential for astrovirus infectivity and the associated mechanisms.

### Insights into astrovirus assembly.

Recombinant human astrovirus proteins, in the form of either VP70 or VP90, with or without the first 70 amino acid residues, have been found to self-assemble into VLPs when overexpressed in mammalian cells (21–23). It is also evident from these studies that the efficiency and accuracy of astrovirus VLP formation were rather low compared to those of HEV as the closest structural homolog. Recombinant HEV CP self-assembles into T=1 or T=3 VLPs, depending on the presence or absence of the N-terminal peptide of the CP (48). However, our results show that astrovirus VP70 expressed in insect cells or E. coli formed predominantly dimers (data not shown). Comparison of the crystal structure of the HEV VLP and the HAstV capsid model shows that the HAstV CP-CP interaction around the 3-fold symmetry axis is substantially weaker, with ∼2,000 Å^2^ of buried surface (S, ∼1,000 Å^2^; P1, ∼1,000 Å^2^; P2, none), compared to ∼3,000 Å^2^ (S, 2,000 Å^2^; P1, 1,000 Å^2^; P2, none) in HEV. Therefore, the lack of efficient assembly of astrovirus VLPs may be due partly to a weak interaction around the 3-fold symmetry axis.

Several approaches may help to enhance the efficiency of astrovirus VLP assembly. For instance, peptide sequences can be either an insertion or conjugated to the N/C terminus of the astrovirus CP to promote trimer formation. Disulfide bond engineering at the trimer interface may also help to stabilize astrovirus CP interactions. Furthermore, molecular modeling based on our VP90^71–415^ capsid model should be able to identify additional mutations that favor trimer formation. The ability to obtain large quantities of VLPs would greatly facilitate detailed characterization of the astrovirus capsid function and the maturation mechanism. Furthermore, development of VLPs that can encapsidate exogenous nucleic acids would allow them to be used as delivery agents of RNA or DNA for different purposes.

### Serotype-related sequence and structural differences.

Near the end of manuscript preparation, the crystal structure of HAstV-1 VP90^71–415^ was reported (49). The structure reported under PDB accession number 5EWN contains two independent HAstV-1 VP90^71–415^ molecules. Superimposition of HAstV-8 VP90^71–415^ onto each of the two HAstV-1 VP90^71–415^ molecules gives average root mean square deviations (RMSDs) of 0.92 Å and 0.90 Å for 324 common C_α_ atoms, which are slightly higher than the average RMSD of 0.83 Å from superimposing the two HAstV-1 VP90^71–415^ molecules. Major variations between the two VP90^71–415^ structures from the two different serotypes are mapped to the following four regions: the S-P1 domain linker, the loop connecting α3 and β12, the loop connecting β13 and β14, and the loop connecting α5 and β15 (Fig. 6a). Additionally, α4 from HAstV-8 is disordered in both subunits of the HAstV-1 structure. Except for the S-P1 domain linker, the other three structurally variable regions all have sequences that are poorly conserved among the eight serotypes (Fig. 6b).

To highlight sequence regions of VP90^71–415^ that are highly variable among the eight HAstV serotypes, HAstV-8 VP90^71–415^ is colored according to the level of sequence conservation (Fig. 6b and c). The S domain is highly conserved, except for the HI loop, which forms a small plateau around the 5-fold symmetry axis and is largely exposed in the viral capsid. The P1 domain contains both highly conserved and poorly conserved regions. The three most poorly conserved regions of P1 are the α3-β12 loop, the β13-β14 loop, and the α5-β15 loop, all of which are completely exposed on the surface of P1. The structural flexibility of these three loop regions is the highest based on temperature factors of the structure. The polypeptide regions from β9 to α3 are also variable but to a lesser degree. The structure of the capsid models shows that this region is partially shielded by other structural elements in the context of the capsid. Our results thus indicate that the presence of the P2 domain does not exclude access to the P1 domain by the host immune system. The top surface of P1, especially the three surface loops mentioned above, may be immunogenic and contain neutralization epitopes recognized by antibodies generated during human astrovirus infection.
